# Behavior of Transition Dairy Cows Managed Outdoors During the Autumn and Spring Calving Seasons

**DOI:** 10.3390/ani15050621

**Published:** 2025-02-20

**Authors:** Daniel Cartes, Rodrigo Held-Montaldo, Pilar Sepúlveda-Varas

**Affiliations:** 1 Departamento Ciencias Clínicas, Facultad de Ciencias Veterinarias y Pecuarias, Universidad de Chile, La Pintana 6640022, Chile; nncartes@uchile.cl; 2 Escuela de Graduados, Facultad de Ciencias Veterinarias, Universidad Austral de Chile, Valdivia 5090000, Chile; rodrigo.heldm@gmail.com; 3 Instituto de Ciencias Clínicas Veterinarias, Facultad de Ciencias Veterinarias, Universidad Austral de Chile, Valdivia 5090000, Chile; 4 Programa de Bienestar Animal, Universidad Austral de Chile, Valdivia 5090000, Chile

**Keywords:** lying time, rumination time, transition, pasture, weather conditions

## Abstract

In pasture-based systems, prepartum dairy cows can be exposed directly to weather conditions during the transition period. We aimed to determine how temperate climatic conditions during spring and autumn calving seasons affect transition dairy cows’ lying and ruminating behavior in outdoor conditions. Behavior and meteorological data were extracted from two external experiments carried out during the spring calving season and the autumn calving season. Mixed models were used to determine the effects of weather conditions on daily lying and rumination behaviors. In the spring calving season, high precipitation reduced the daily lying time, and the wind speed decelerated this effect both in the pre-and postpartum period. High precipitation combined with low air temperature or wind speed favored the rumination time in pre- and postpartum periods. In the autumn calving season, lying time was reduced as air temperature and wind speed increased during the prepartum period, and solar radiation increased in the postpartum period. The rumination time was reduced under warm conditions (high temperature/solar radiation) in pre- and postpartum periods. These findings suggest that under a temperate climate, the weather of both calving seasons has important implications on behaviors associated with comfort, such as lying and rumination.

## Simple Summary

In pasture-based dairy systems, the farmers adjust the calving season to short periods of the year to match the higher lactation requirements with the greater availability of pasture as a food source. These periods coincide with adverse climatic conditions of southern Chile that can affect the comfort and welfare of the transition cows. Therefore, we aimed to determine how temperate climatic conditions during two calving seasons (spring and autumn) affect transition dairy cows’ lying and rumination behaviors in outdoor conditions. In the spring calving season, rainy conditions reduced lying time in the pre- and postpartum periods. Also, rainfall combined with low temperatures or wind speed increased rumination time in both periods. In the autumn calving season, lying time was reduced as solar radiation increased during pre and postpartum periods, and wind mitigated this effect. The rumination time was reduced under warm conditions in pre and postpartum periods. Our results show that the interaction of different weather conditions modulates essential behaviors such as lying and rumination of transition dairy cows.

## 1. Introduction

In many pasture-based systems, including southern Chile, seasonal outdoor calving is strategically managed to occur during late summer to early autumn or late winter to early spring (the autumn and spring calving seasons, respectively) to maximize pasture availability to support lactation and reduce feeding costs [1]. However, adverse weather conditions can significantly challenge the welfare of transition dairy cows. For example, cows calving in summer often face high temperatures, elevated humidity, and intense solar radiation [2], while those calving in winter are subjected to low temperatures, precipitation, wind [3,4], and muddy underfoot conditions [5,6].

In warm weather, cows employ various behavioral strategies to prevent increased body temperature. These coping mechanisms encompass seeking shade [7,8,9,10,11], adjusting their feeding/grazing times to cooler times of the day [7,12], increasing their water intake and spending more time around water sources [11,13], reducing their lying time [8,11,14], decreasing their activity and movement [9,15], and reducing their rumination time [16,17]. Research conducted with prepartum-housed Holstein cows has also indicated their adaptation to heat stress by lowering daily lying [12] and rumination times [18]. Studies have shown that biological functioning can be negatively affected during heat stress, a welfare concern in many cases. For example, as the temperature–humidity index (THI) rises, there is a decrease in dry matter intake (DMI) in lactating dairy cows, which reduces milk production [15,19]. This lower DMI could be particularly important during the prepartum period since DMI depression before parturition is linked to several postpartum metabolic disorders [20,21].

In cold, wet, and windy weather, outdoor-managed cows make efforts to adapt by altering their behavior to preserve body temperature [22,23]. By decreasing lying time [6,11,24,25] and lying down with their legs tucked under their bodies [24], cows may reduce conductive heat loss as much as possible. Dairy cows also seek protection in periods of inclement weather [11], and evidence suggests that having access to a shelter increases lying time during the prepartum period and reduces adipose mobilization [26]. Research has also shown that temporarily managing dairy cows on wet and muddy surfaces harms their welfare. For instance, experimental works have shown that muddy surfaces severely reduced lying times by 50 to 75% compared to dry surfaces [25]. In addition, evidence indicates that lactating and dry cows managed outdoors during winter months reduce their lying time on days of rainfall [6,27] and show rebound responses the day after rainfall, indicating that the motivation to rest is not fulfilled on such days [6]. Hendriks et al. [3] observed that the lying patterns of transition dairy cows are inversely related to air temperature and precipitation under winter conditions in New Zealand. However, there is limited information on rumination behavior, and no studies have linked these findings to the prepartum or postpartum periods.

Most of the studies on dairy cattle’s exposure to inclement weather have been carried out on lactating or dry cows. However, little research has focused on the weeks around calving, which is the most critical time for cow health, survival, and the overall profitability of lactation. Thus, our study aimed to determine the effect of weather on the lying and rumination behavior of outdoor-managed transition dairy cows during the autumn and spring calving seasons in a temperate climate.

## 2. Materials and Methods

This study was conducted as part of a larger investigation undertaken to describe behavioral changes in transition dairy cows during the winter–early spring calving season of 2018 (July to October) and summer–early autumn calving season of 2019 (February to April; Southern Hemisphere) at the Austral Agricultural Research Station of the Austral University of Chile (Valdivia, Region de Los Ríos, Chile; 39°46′42 S, 73°13′38 W). Animals were cared for according to the Animal Care Ethics Committee of Universidad Austral de Chile (Protocol N°328, 2017).

Housing, diet, and general management were as described by Held-Montaldo et al. [4]. Before enrolling in this study, cows had been managed outdoors on pasture paddocks (mixture of grasses and legumes) during the far-off dry period. Approximately three weeks before the expected calving date, cows were moved to a 1.7 ha paddock without pasture (i.e., a bare soil surface paddock), remaining until calving. The group of prepartum animals was dynamic, entering the study based on the expected calving date and leaving the group once they had calved. After calving (0 or 1 DIM), cows were moved to one mixed parity lactating group and were managed daily in a rotational grazing method under pasture paddocks of 3 to 5 ha.

### 2.1. Study Design and Sample Size

The study design was a retrospective observational study. Records were collected from a convenience sample of 103 Holstein multiparous dairy cows that calved during the spring (*n* = 57) and autumn (*n* = 46) seasons [4]. Cows that presented any clinical disorder (dystocia, retained placenta, hypocalcemia, lameness, mastitis, or metritis) during the transition period (from 3 weeks before calving until 3 weeks after calving) were excluded (spring = 38 cows; autumn = 29 cows). Thus, 36 clinically healthy multiparous cows contributed to the presented study, distributed between the spring (*n* = 19) and autumn (*n* = 17) calving seasons.

### 2.2. Lying and Rumination Behavior

To measure daily lying behavior from 3 weeks before to 3 weeks after calving, electronic data loggers (HOBO^®^ Pendant G Acceleration Data Logger, Annapolis, MA, USA) were used. Loggers were attached to the lateral side of one of the hind legs of each cow with a vet wrap approximately 30 days before the estimated calving date. Every ten days, the devices were replaced and reattached to the opposite leg to alternate them, allowing for data downloading and preventing data loss from battery depletion. Each logger was programmed to record position changes at 1 min intervals for consecutive hours. This information was used to determine whether the cow was standing or lying and subsequently used to calculate daily lying time, number of lying bouts (frequency of transitions from standing to lying positions), and duration of lying bouts as validated for dairy cows by Ledgerwood et al. [28]. Recorded data during the day the loggers were changed were removed.

Rumination time was recorded using an individual rumination logger on a neck collar. This automatic system (Hr-Tag^®^, SCR Engineers Ltd., Netanya, Israel) was validated for dairy cows by Schirmann et al. [29] and continuously records rumination time every 2 h. The collars were put on at the same time as the lying data logger and were kept up to 3 weeks after calving. Tag information was transferred to the control unit through radio frequency, allowing backup from the control unit. The data in the unit control were downloaded to the database weekly. The entire day was discarded if the system did not record one 2 h interval.

### 2.3. Weather Data

Data were collected from the Institute of Agricultural Research (INIA) Weather Station (www.agromet.cl, accessed on 11 September 2019), located approximately 800 m from the paddocks where the animals were managed. The weather factors were recorded every 1 h during each season, including air temperature (°C), relative humidity (%), precipitation (mm), solar radiation (w/m^2^), and wind speed (m/s). To characterize the weather conditions throughout the autumn and spring calving seasons, average values were computed based on hourly data, except precipitation, for which estimates were made only on rainy days.

### 2.4. Statistical Analysis

Weather and behavior data (lying time, lying bouts, duration bouts, and rumination time) were summarized by day in the prepartum (day −21 to day −3 relative to calving) and postpartum (day 3 to day 21 relative to calving) period. Data between days −2 and 2 relative to calving were excluded from the analysis due to the influence of calving on lying and rumination behaviors [30,31,32]. The analysis was carried out using R language [33] and R packages lme4 [34] and lmerTest [35]. In the first approach, behavioral values of each period relative to calving (pre- and postpartum) were compared between the two calving seasons using mixed models, where the “cow” was included as a random effect. Data were presented as least squares means and standard error (SE).

Afterward, daily weather data were combined with daily cow behavior data for each calendar day, and mixed models were used to determine the effects of weather variables on daily behaviors in each period (pre and postpartum period) on two calving seasons. This analysis considered the cow as a random effect. Air temperature, wind speed, precipitation, solar radiation, and interactions were considered fixed effects during the spring calving season, while air temperature, solar radiation, wind speed, and interactions were considered continuous covariates during the autumn calving season. Relative humidity was not considered in the analysis because it had a high correlation with solar radiation (Pearson correlation coefficient: −0.81, *p* < 0.001). Based on the lowest Akaike’s Information Criteria AIC and *p*-value of <0.05 of the overall model, it was decided whether additional climate factors improved the model fit. All model residuals were visually assessed and were normally distributed. Regression coefficients and their SE were calculated and reported.

## 3. Results

### 3.1. Weather Data

During the autumn and spring calving seasons, a wide range of environmental conditions were recorded (Table 1). Precipitation was not reported in the autumn season because it averaged <1 mm/day throughout the study period.

### 3.2. Daily Lying and Rumination Behavior

**Figure 1:**
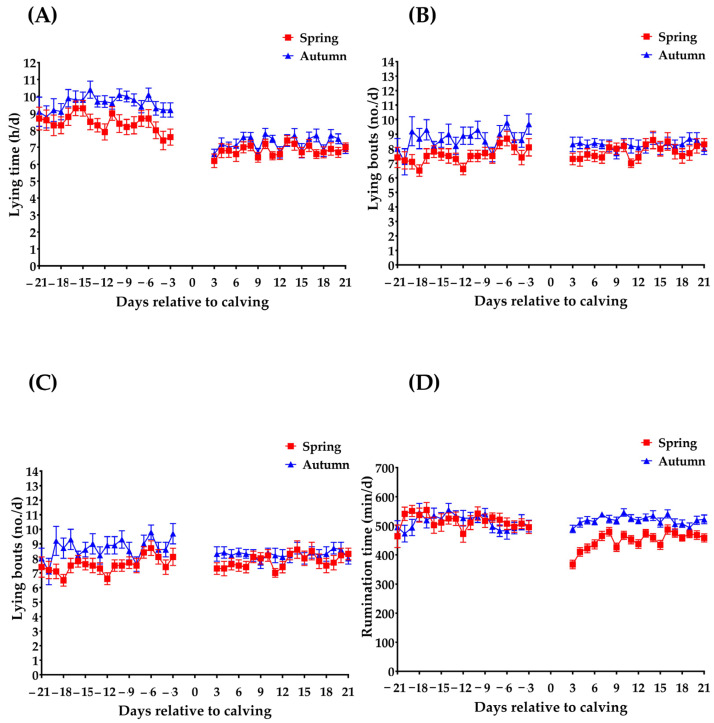
Daily lying time (h/d; (**A**)), lying bouts (no./d; (**B**)), lying bouts duration (min/bout; (**C**)), and rumination time (min/d; (**D**)) of clinically healthy multiparous dairy cows during the prepartum and postpartum period over the spring (*n* = 19) and autumn (*n* = 17) calving season. Values are presented as means ± SE.

Details about the mean values recorded for lying and rumination behavior during each season and periods relative to calving can be found in Table 2. Lying behavior was different between calving seasons; cows that calved during spring presented a lower lying time during the prepartum period compared to the lying time of the prepartum cows in the autumn season (approximately 1 h, *p* = 0.002). During the postpartum period, the lying time of cows that calved in the spring season tended to be lower (approximately 0.6 h, *p* = 0.056) compared with cows that calved during the autumn season (Figure 1A). Spring-calved cows presented fewer transitions from standing to lying position during the prepartum period (*p* ˂ 0.001) and similar transitions during the postpartum period (*p* = 0.055) compared with their counterparts during autumn (2 and 1 bout, respectively; Figure 1B). Lying bouts duration during spring was 9 min longer during the prepartum period than in autumn (*p* = 0.004), with no differences in the postpartum period (*p* = 0.60; Figure 1C).

Rumination behavior was also different between both calving seasons. The spring prepartum rumination time was 49 min higher than the values recorded during autumn (*p* = 0.04; Figure 1D). However, an inverse relationship was found during the postpartum period; spring-calved cows tended to ruminate almost 40 min less than their autumn counterparts (*p* = 0.09; Figure 1D).

### 3.3. Effect of Weather on Prepartum Lying and Rumination Behavior

Model outputs for the association between weather conditions and behavior in prepartum cows can be found in Table 3. During the spring calving season, prepartum cows tended to lie down less and had fewer lying bouts on days with rainfall (*p* < 0.001). However, this decrease was less pronounced on days with higher wind speeds (precipitation x wind speed interaction; *p* < 0.001) and more pronounced on days with increased solar radiation (precipitation x solar radiation interaction; *p* < 0.001). Overall, higher ambient temperatures (*p* < 0.01), precipitation (*p* < 0.001), and windy conditions (*p* < 0.05) reduced mean lying bout duration in prepartum cows. Also, an interaction was noted between air temperature and wind speed, with wind speed reducing the effect of ambient temperature by prolonging the duration of bouts. Precipitation was associated with increased total daily rumination time (*p* < 0.01), and more air temperatures reduced this effect due to an interaction between both weather variables (*p* < 0.01).

During autumn, prepartum cows exhibited a decrease in their daily lying time as temperature, wind speed, and solar radiation increased (*p* < 0.001). An interactive effect was also observed; the impact of solar radiation was less pronounced when wind speed was higher (*p* < 0.001). Cows transitioned less frequently from standing to lying position with higher air temperature (*p* < 0.05) and wind speed (*p* < 0.001), while lying bout duration was increased under windy conditions (*p* < 0.001) and decreased with high solar radiation. High air temperature (*p* < 0.001) and solar radiation (*p* < 0.001) were both found to decrease daily rumination time. Additionally, an interaction between these two variables was noted: when one increased, it lessened the effect of the other.

### 3.4. Effect of Weather on Postpartum Lying and Rumination Behavior

Model outputs for the association between weather conditions and behavior in postpartum cows can be found in Table 4. During the spring calving season, rainfall reduced the lying time of cows (*p* < 0.001), which was countered by windy conditions due to an interaction (*p* < 0.01). Also, under these winter conditions, a high air temperature and solar radiation increased the lying time (*p* < 0.01). Lying bouts were reduced with the increase in rainfall, and more wind speed reduced this effect due to an interaction between both climatic variables (*p* < 0.001). Similarly, precipitation reduced the mean lying bout duration (*p* < 0.01), while solar radiation increased the duration of each bout. Rumination time was increased in the presence of rainy conditions (*p* < 0.001). However, under high rainfall levels, the wind reduced rumination time due to an interaction (*p* < 0.01).

In the autumn season, a few variables influenced lying and ruminating behaviors during the postpartum period. Daily lying time was reduced only by increased solar radiation (*p* < 0.001), and no interactions were observed between weather measures. Lying bouts were affected by air temperature (*p* < 0.001), wind speed (*p* < 0.001), and solar radiation (*p* < 0.001), where a higher temperature and solar radiation decreased the number of lying bouts due to an interaction. Higher air temperatures shortened each bout’s duration, while increased wind speed prolonged it without interaction between the two variables. The rumination time was only affected by solar radiation, where as the radiation increased, the rumination time was reduced (*p* < 0.05), and no interactions were observed.

## 4. Discussion

This study examines the effect of different climatic conditions on the lying and rumination behavior during two different calving seasons in transition dairy cows under a pasture-based system in a temperate region in southern Chile. Our results showed that dairy cows managed outdoors may display behavioral differences between calving seasons within a year, demonstrating an association with climatic variables such as rainfall, ambient temperature, wind, and solar radiation.

The average daily prepartum lying time recorded during the autumn calving season was 1 h higher than spring-calved cows (10.4 h/d vs. 9.4 h/d, respectively). Similarly, during the postpartum period, spring-calved cows had lower lying times than autumn-calved cows (~0.6 h/d). Our results showed that differences in lying time among calving seasons were related to differences in weather conditions; during the spring calving season, cows during the pre and postpartum period were exposed to high rainfall (range 0–130 mm/d) compared with the autumn calving season (0 mm/d). Previous studies have also found that lying time is affected by rainfall during winter months [3,27,36]. Rainfall increases the chances of cows being exposed to wet and muddy surfaces, which have been described to impair lying behavior in mid-lactation and dry cows [37,38,39]. Although we did not evaluate the mud accumulation between both seasons, it is reasonable to consider that the surface condition during autumn was drier, given the decline in rainfall compared to the spring.

We observed that rainfall conditions substantially compromised the lying time in both periods relative to calving. This effect increased with higher solar radiation during the prepartum period, while stronger wind diminished the impact of precipitation in both the pre-and postpartum periods. The effects of rainfall have been early reported by Tucker et al. [24], Webster et al. [40], and Schütz et al. [41], finding reductions of more than 50% in the time of lie down of cows under simulated winter conditions, due to the formation of an uncomfortable wet surface for rest. In the natural environment of our study, the increase in precipitation could reduce their insulation capacity, favoring the loss of body heat [22]. This loss of body heat is favored by increased wind speed because heat is carried away from the body faster, driving down both the skin and internal body temperatures [42,43]. For this reason, we believe that in rainy conditions, increased wind causes cows to spend more time lying down. This behavior reduces the body surface area exposed to the wind, which in turn decreases heat dissipation to the environment. On the contrary, solar radiation increases the heat gain of the cows and the surroundings [44]. For this reason, under a rainy scenario, the cows could increase standing time (or reduce lying time) to allow a greater body surface area to be exposed to direct and indirect radiation.

In the current study, prepartum spring-calved cows presented fewer lying bouts than the cows followed during the autumn season. During the spring season, the reduction in the frequency and duration of lying bouts was observed mainly under rainy and windy conditions, while the increase in wind speed decelerated this effect, just as it occurred with the cows’ lying time. However, rising air temperatures mitigated the impact of wind speed on the prepartum lying bouts. In pasture-based systems, increased rainfall and animal movement often result in wet and muddy resting surfaces [4]. As observed in previous studies [3,25,27,45], these conditions result in a lower frequency and shorter lying episodes, indicating a reluctance in the transition cows to lie down and persist on these surfaces. The slowdown in the effect of wind-driven precipitation on lying bouts is similar to that observed on lying time. This can be explained by an increased number of transitions to lying, which minimizes the surface area exposed to cold air, especially when humidity from precipitation decreases the insulation capacity [3,22].

The daily mean temperature, wind speed, and solar radiation were relevant weather variables in the autumn calving season. In the prepartum period, increasing warm environmental conditions, such as higher air temperature, solar radiation, and higher wind speed, reduced the cows’ daily lying time. Similar experiences were reported in lactating and dry cows under temperate conditions of the Netherlands, where standing time showed the inverse effect of temperature and THI, which began at low-temperature thresholds (12 to 16 °C) [14]. Under heat conditions, the standing posture exposes a larger skin surface area to the surrounding air than a reclining posture. Consequently, a greater heat dissipation rate can be achieved [46,47]. On the other hand, more solar radiation reduced lying time in both periods, but during prepartum, this effect was mitigated by the increase in wind speed. Part of this response agrees with responses found by Tucker et al. [8] and Schütz et al. [11] in dairy cows under temperate conditions. Solar radiation increases the heat load of cows and their surroundings [44]. By being upright, the cows may avoid lying on surfaces with temperatures higher than their body surfaces, thereby avoiding heat gain by conduction [42,44]. The mitigating effect of wind under warm conditions, such as high solar radiation, is in line with our expectations and what was reported in previous studies with cows under free-stall systems and with the use of cooling systems [48] or fans [49]. The wind increases, and heat is carried away from the body faster, driving down both the skin and internal body temperatures [42,43], expecting the cows to maintain or increase the laydown position.

In the autumn calving season, an increase in wind speed reduced the number of daily lying bouts and increased the duration of the bouts during the pre-and postpartum period. Also, in this season and under warm conditions (higher air temperature and/or solar radiation), the cows had shorter lying bouts. These changes in the bout numbers and the duration of each bout may be directly related to the rate of gain or loss of core body temperature (CBT), as suggested by Norlund et al. [42]. These authors reported that lactating cows kept in free-stall barns had shorter lying periods as the ambient conditions were warmer and the rate of CBT gain increased. However, in the presence of methods that favor heat dissipation, like more wind speed or other abatement strategies, the CBT gains in lying cows could be reduced [42,50], extending the duration of each lying bout, just like in our work with natural wind.

### Rumination Behavior

In the present study, the rumination time of transition cows during the spring season was, on average, 549 min/d and 483 min/d during the pre-and postpartum periods, respectively. Also, during the autumn season, cows ruminated on average 500 min/d and 520 min/d in the pre-and postpartum periods, respectively. These values are comparable to the 540 min/d reported by Matamala et al. [51] for cows kept in outdoor conditions during the prepartum period. However, recently, Iqbal et al. [52], in the pasture-based system in New Zealand, found a great variability of rumination times in different breeds of cows, number of lactations, and year season, ranging from low values of 313 min/d to 469 min/d.

Weather conditions can be relevant in explaining the variability of rumination times in each season. During the spring calving season, rainy days during pre or postpartum generated more rumination times, but the increase in air temperature (prepartum) or wind speed (postpartum) slowed down this effect. These results partially agree with those reported by Redbo et al. [53] in dairy heifers and Graunke et al. [54] in beef cattle under Swedish winter conditions. In these studies, it was observed that the cattle increased the time spent ruminating in protected areas under rainy conditions. The present work had no protection areas, but during several rain episodes, the animals were observed ruminating, standing up, and grouped, which has been suggested as an attempt to protect and preserve body heat [55]. Also, the rumination process is highly thermogenic and could be essential to mitigate winter conditions [43]. This could explain the reduction in rumination time with the rise in temperature under rainy conditions.

Contrary to the spring season, warm days in the autumn calving season reduced the rumination time. The increased air temperature and high solar radiation reduced the rumination time. In contrast, during the postpartum period, the rumination time was reduced only by the effect of solar radiation. Similar effects have been described by Müschner-Siemens et al. [17] in lactating cows exposed to moderate heat stress conditions in Germany or Hut et al. [14] in the Netherlands, where the rumination time of lactating cows decreased by 9 min/d at a THI ≥ 72 (mild heat stress) under automatic milking systems (AMS) and 5 min/d at THI of ≥56 (Thermoneutral Zone) in dry cows. The fermentation in the rumen increases the metabolic heat [56,57], so reducing ruminal activity under warm conditions could be a mechanism to maintain or avoid increases in the body temperature. In this sense, periparturient cows exposed to cooling methods or lactating dairy cows with access to shade increased rumination time [58].

## 5. Conclusions

During the spring and autumn calving seasons, climatic conditions affected transition cows’ lying and rumination behaviors outdoors. Among the weather variables evaluated in the spring calving season, daily precipitation was the most critical variable in lying and rumination behaviors in both the pre-and postpartum periods. The increase in rainfall combined with other variables was associated with reduced daily lying times and increases in daily rumination time, in an attempt to adapt to cold conditions and wet surfaces for rest. During the autumn calving season, the increase in solar radiation combined with other weather variables was associated with less daily lying and rumination times, as alternatives to promote heat dissipation and reduce the heat caused by ruminal fermentation. These findings suggest that under a temperate climate, transition cows of both calving seasons are affected by different weather variables, which must be considered to improve well-being during the transition period in a pasture-based system.

## Figures and Tables

**Table 1:** Daily weather conditions experienced by dairy cows over the prepartum and postpartum period during the autumn (February to April) and spring (July to October) calving season in southern Chile.

Period	Weather Variable ^1^	Autumn ^2^		Spring	
		Mean ^2^ (2160 Data)	Range	Mean ^2^ (2880 Data)	Range
Pre	Air temperature (°C)	15.1	9.5–25.1	7.8	1.8–12.6
	Relative humidity (%)	71.9	34.8–93.5	88.5	68.8–99.7
	Wind speed (m/s)	4.7	0.5–13.0	5.4	0.2–18.7
	Solar radiation (W/m^2^)	217.6	51.9–325.4	90.9	15.9–207.1
	Precipitation (mm/d) ^3^	0	0	10.2	0.0–130.0
Post	Air temperature (°C)	13.5	6.5–23.6	8.6	1.8–14.3
	Relative humidity (%)	77.2	34.8–97.0	86.5	68.5–99.7
	Wind speed (m/s)	4.5	0.0–15.0	5.5	0.9–18.7
	Solar radiation (W/m^2^)	176.1	16.0–307.4	120.8	15.9–294.5
	Precipitation (mm/d) ^3^	0	0	8.6	0–130.0

^1^ All the weather variables were recorded continuously every 1 h; ^2^ means were calculated by season using the 1 h records of each parameter; ^3^ mean of the precipitation was calculated considering only the days it rained.

**Table 2:** Comparison of lying time, lying bouts, lying bouts duration, and rumination time (LSM ± SE) during the prepartum and postpartum period recorded during the autumn (*n* = 17) and spring calving season (*n* = 19) in clinically healthy multiparous dairy cows.

Period ^1^	Behavior	Spring		Autumn		*p*-Value
		Mean	SE	Mean	SE	
Pre	Lying time (h/d)	9.4	0.2	10.4	0.1	0.002
	Lying bouts (no./d)	7.0	0.2	9.0	0.1	˂0.001
	Bout duration (min)	81.3	1.4	72.4	1.4	0.004
	Rumination time (min/d)	549	6.3	500	6.4	0.040
Post	Lying time (h/d)	6.7	0.1	7.3	0.1	0.056
	Lying bouts (no./d)	7.0	0.1	8.0	0.1	0.055
	Bout duration (min)	61.4	1.1	59.9	1.0	0.600
	Rumination time (min/d)	483	5.5	520	4.0	0.090

^1^ Prepartum: day −21 to −2; Postpartum: day +2 to +21.

**Table 3:** Effect of the weather conditions on prepartum lying and rumination behavior during spring (*n* = 19) and the autumn calving season (*n* = 17) and in clinically healthy multiparous dairy cows.

Behavior	Weather Variables ^1^	Spring			Autumn ^2^		
		Estimate	SE	*p*-Value	Estimate	SE	*p*-Value
Lying time (h/d)	Intercept	10.7	0.5	<0.001	13.7	0.65	<0.001
	T	-	-	-	−0.2	0.04	<0.001
	WS	−0.01	0.03	0.69	−0.25	0.06	<0.001
	P	−0.18	0.02	<0.001	-	-	-
	SR	−0.005	0.001	0.05	−0.008	0.0002	<0.001
	P × WS	0.01	0.002	<0.001	-	-	-
	P × SR	−0.001	0.001	<0.001	-	-	-
	SR × WS	-	-	-	0.001	0.0002	<0.001
Lying bouts (no./d)	Intercept	8,9	0.5	<0.001	11.2	0.8	<0.001
	T	-	-	-	−0.13	0.5	<0.05
	WS	0.008	0.03	0.98	−0.21	0.04	<0.001
	P	−0.13	0.02	<0.001	-	-	-
	SR	0.006	0.006	0.20	-	-	-
	P × WS	0.007	0.001	<0.001	-	-	-
	P × SR	−0.001	0.0002	<0.001	-	-	-
Bout duration (min)	Intercept	88.7	5.07	<0.001	74.8	4.3	<0.001
	T	−1.89	0.61	<0.01	-	-	-
	WS	−2.13	1.03	<0.05	1.7	0.3	<0.001
	P	−0.37	0.08	<0.001	-	-	-
	SR	-	-	-	−0.06	0.01	<0.001
	T × WS	0.23	0.10	<0.05	-	-	-
Rumination time (min/d)	Intercept	380.4.0	28.5	<0.001	906.1	94.5	<0.001
	T	2.6	2.0	0.4	−28.4	7.1	<0.001
	P	5.38	1.5	<0.01	-	-	-
	SR	-	-	-	−1.3	0.3	<0.001
	P × T	−0.5	0.1	<0.01	-	-	-
	T × SR	-	-	-	0.07	0.02	<0.05

^1^ Air temperature (T): °C; wind speed (WS): m/s; precipitation (P): mm; solar radiation (SR): W/m^2^. ^2^ The effect of P (precipitation) was not considered in the analysis of autumn data due to the lack of this meteorological condition. (-) The dash symbol represents a significant variable or interaction included in the best-fitted model in one calving season but not in the other.

**Table 4:** Effect of the weather conditions on postpartum lying and rumination behavior during spring (*n* = 19) and the autumn calving season (*n* = 17) and in clinically healthy multiparous dairy cows.

Behavior	Weather Variables ^1^	Spring			Autumn ^2^		
		Mean	SE	*p*-Value	Mean	SE	*p*-Value
Lying time (h/d)	Intercept	5.8	0.4	<0.001	6.3	0.6	<0.001
	T	0.15	0.04	<0.01	-	-	-
	WS	−0.01	0.05	0.80	-	-	-
	P	−0.12	0.01	<0.001	-	-	-
	SR	0.01	0.003	<0.001	−0.004	0.00	<0.001
	P × WS	0.005	0.001	<0.01	-	-	-
Lying bouts (no./d)	Intercept	9.24	0.3	<0.001	6.7	0.6	<0.001
	T	-	-	-	0.4	0.09	<0.001
	WS	0.002	0.03	0.94	−0.27	0.06	<0.001
	P	−0.16	0.02	<0.001	-	-	-
	SR	−0.004	0.001	<0.05	0.02	0.005	<0.001
	P × WS	0.01	0.002	<0.05	-	-	-
	SR × T	-	-	-	−0.002	0.0004	<0.001
Bout duration (min)	Intercept	50.6	2.6	<0.001	82.0	9.0	<0.001
	T	-	-	-	−1.9	0.6	<0.01
	WS	−0.53	0.2	<0.05	0.8	0.2	<0.05
	P	−0.29	0.1	<0.01	-	-	-
	SR	0.06	0.01	<0.001	-	-	-
Rumination time (min/d)	Intercept	391.9	20.8	<0.001	607.0	77.0	<0.001
	WS	2.4	1.4	0.11	-	-	-
	P	1.9	0.7	<0.05	-	-	-
	SR	-	-	-	−1.8	0.7	<0.05
	P × WS	−0.2	0.06	<0.01	-	-	-

^1^ Air temperature (T): °C; wind speed (WS): m/s; precipitation (P): mm; solar radiation (SR): W/m^2^. ^2^ The effect of P (precipitation) was not considered in the analysis of autumn data due to the lack of this meteorological condition. (-) The dash symbol represents a significant variable or interaction included in the best-fitted model in one calving season but not in the other.

## Data Availability

Dataset available on request from the authors.
